# Kinetics and modeling of growth and lactic acid production in *Gundruk*, a Himalayan fermented vegetable dish

**DOI:** 10.1002/fsn3.1854

**Published:** 2020-09-01

**Authors:** Arjun Ghimire, Ajit Kumar Sah, Ranjana Poudel

**Affiliations:** ^1^ Department of Food Technology Central Campus of Technology Dharan Nepal; ^2^ Department of Food Technology Dharan Multiple Campus Dharan Nepal

**Keywords:** lactic acid fermentation, microbial growth, modeling

## Abstract

*Gundruk* is a fermented green leafy vegetable product prepared from fresh leaves of local vegetables called *Rayo*—*sag* (*Brassica campestris*), mustard (*Brassica juncea)*, and cauliflower *(Brassica oleracea)* indigenous to the Nepali people. Fresh *gundruk was* prepared in a glass jar by fermenting the *Brassica juncea* leaves anaerobically for 16 days and the changes in biomass, lactic acid, and pH were evaluated after every 24 hr. The viable cell count increased from 6.03 × 10^4^ cfu/g to 9.55 × 10^8^ cfu/g after 3 days and then decreased gradually to remain constant after 8 days with 6.31 × 10^7^ cfu/g until the end of fermentation. The lactic acid increased by about 12.58 times in 12 days and remained constant for the rest of the fermentation period. Unlike this, pH decreased from 6.59 to 3.71 on the 9th day of fermentation and then increased slightly till the last day of fermentation. The data obtained were fitted to two most widely accepted microbial growth models: Modified Gompertz, and Logistic model and three well‐known lactic acid production models: Luedeking‐ Piret, Monteagudo et al., and Balannec et al. model for lactic acid fermentation. Based on nonlinear regression analysis, Modified Gompertz, and Monteagudo et al. model gave a better fit to describe microbial growth and lactic acid production, respectively. The growth‐associated and non‐growth‐associated coefficients were determined to be 0.1104 and 0.0042, respectively, using Monteagudo et al. model. The findings revealed that lactic acid production in *gundruk* is a mixed type.

## Graphical abstract

- The lactic acid increased by about 12.58 times in 12 days and remained constant for the rest of the fermentation period.
- The exponential increase in the LAB population was significant until 3rd day detecting at the level of almost 9.55 × 108 cfu/ml.
- Modified Gompertz model gave a better fit for LAB growth while the Monteagudo et al. model was well fitted for lactic acid production.

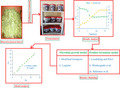

**Nomenclature**
[HL]: Undissociated lactic acid concentration (g/L)
BJL: Brassica juncea leaves
BM: Beet molasses
LAB: Lactic Acid Bacteria
LB: Lactobacillus brevis
LD: Lactobacillus delbrueckii
LH: Lactobacillus helveticus
LP: Lactobacillus plantarum
MRS: De Man, Rogosa and Sharpe
N: Cell concentration (cfu/g)
N_0_: Initial cell concentration (cfu/g)
P: Product concentration (g/L)
PDA: Potato Dextrose Agar
P_max_: Maximum lactic acid concentration (g/L)
t: Fermentation time
VJ: Vegetable juice
WP: Whey permeate
X: Cell concentration (g/L)
YE: Yeast extract
YM: Yeast Malt

## 1 INTRODUCTION

Gundruk is an acidic dried vegetable product indigenous to the Nepali residing in the mountainous regions of Nepal, India, and Bhutan. The fresh leaves of local vegetables called *Rayo*—*sag* (*Brassica rapa* subsp. *campestris* variety *cuneifolia*), mustard (*Brassica juncea)*, and cauliflower (*Brassica oleracea)* are wilted in sun and shredded, crushed, and squeezed tightly into an earthen container or jar, which is made airtight to favor the creation of anaerobic environment. The container is then made warm and fermentation occurs naturally for about 10 days. Unlike *kimchi* and sauerkraut, fermented *gundruk* is allowed to dry in sun for 3–4 days before consumption and can be preserved for more than 2 years at room temperature. *Lb. fermentum*, *Lb. plantarum*, *Lb. casei*, *Lb. casei* subspecies *pseudoplantarum,* and *P. pentosaceus* have been isolated from *gundruk* (Tamang et al., 2005). *Gundruk* is sold in all local markets and eaten as soup or pickles (Tamang et al., 2012). *Gundruk* fermentation is initiated by *Lb. cellobios* and is followed by *P. pentosaceus* and finally by *Lb. plantarum*, *Lb. casei*, and *Lb. casei* subspecies *pseudoplantarum*. A heterofermentative *Lb. cellobiosus* produces carbon dioxide and ethanol making favorable anaerobic environs for the lactic acid bacteria (Karki, Okada, Baba, Itoh, & Kozaki, 1983). Some LAB isolated from *gundruk* showed strong acidification, antimicrobial properties, abilities to degrade anti‐nutritive factors, and probiotic character (Tamang, Tamang, Schillinger, Guigas, & Holzapfel, 2009). *Gundruk* is considered as a good appetizer (Tamang, 2010).

*Gundruk* production techniques have been restricted to the family level and transferred from mother to daughter, where the quality attributes are judged by the typical flavor and acidic taste. Naturally, the sugar in the vegetable is fermented to lactic acid by acid‐forming bacteria increasing the acidity which is the measuring index of *gundruk* quality (Karki et al., 1983). Lactate fermentation is one of the popular preservation methods and still serves as substitutes where refrigeration and other means are not available for the safekeeping of food (Holzapfel, 2002). Lactic acid bacteria are extensively used as starter cultures for fermentation thereby, increasing the shelf life of foods and offering characteristic organoleptic qualities (Esteban‐Torres et al., 2015). Apart from preservation, fermented foods include advantages of enhancing sensory attributes, increased digestibility, and improving nutritional and pharmacological qualities (Jeyaram, Romi, Singh, Devi, & Devi, 2010). Regarding food applications, bacteriocins from LAB have characteristics benefit over standard antibiotics since, in contrast to the later, LAB bacteriocins are commonly viewed as food‐grade because of its association in food fermentation that dates back to precedent days. In reality, the U.S. Food and Drug Administration (FDA) has considered LAB and its by‐products to be Generally Regarded as Safe (GRAS) for human consumption (Lahtinen, Ouwehand, Salminen, & Wright, 2011).

The kinetic models play an important role in monitoring and predicting the fermentation process which contain kinetics of substrate utilization, growth, and product formation. According to this model, cell growth models can be divided into unstructured and structured types. Most of the available mathematical models for the lactic acid fermentation process are unstructured that are the simplest and take the cell mass as a uniform quantity without internal dynamics whose reaction rate depends only upon the conditions in the liquid phase of the reactor. This model contains a small number of parameters which can easily be estimated based on steady‐state experiments and open‐ended (Lewis & Young, 1995), and can rather easily be extended to describe more complex systems (Esener, Roels, & Kossen, 1983).

The kinetics of microbial growth in lactic acid fermentation has been studied by Mercier, Yerushalmi, Rouleau, and Dochain (1992) and Norton, Lacroix, and Vuillemard (1994). They used the logistic model that expresses the relationship of the growth rate and two kinetic parameters, such as the maximum specific growth (μ_m_) and the biomass concentration. Sharma and Mishra (2014) fitted the modified Gompertz equation to the logarithm of the cell concentration to determine the maximum specific growth rates, lag phase, and maximum cell numbers of *L. plantarum* in various media. Three types of fermentation can be distinguished such as growth‐associated product formation, mixed growth‐associated product formation, and non‐growth‐associated product formation (Moser, 1985). Many researchers used the mixed growth‐associated product formation for lactic acid production kinetics which suggests that the product (P) formation rate depends on the growth rate and the cell concentration. The kinetics model for lactic acid production on beet molasses using *L. delbrueckii* was proposed by Monteagudo, Rodríguez, Rincón, and Fuertes (1994). Using the model given by Luedeking and Piret (1959), it improved by the addition of a term indicating the dependence of the rate of lactic acid production on inhibitor concentration of the lactic acid. According to this equation, the lactic acid formation rate will become zero when lactic acid concentration approaches its maximum concentration (Suscovic, 1992). Amrane and Prigent (1994) noted that the beginning of the production is accurately described by Luedeking and Piret relation, namely for significant values of the specific growth rate. However, almost half of the lactic acid is produced during the deceleration and the stationary growth phases, whereas the specific growth rate tends toward the zero value. Since the undissociated form of lactic acid is the main growth inhibitor, Balannec, Bouguettoucha, and Amrane (2007) considered the undissociated form of the product instead of its total amount. The inhibitory term was added to the non‐growth‐associated part of the production, to account for cessation of production in case of culture without pH control or at acidic pH.

The present study is a preliminary effort toward finding the possibility of improving the traditional technology to commercialize using the best fermentation condition for the preparation of *gundruk* using an unstructured mathematical model. Here, we analyze the kinetics of biomass production, pH, and lactic acid production during fermentation of *gundruk* and the obtained results can assist in process control and the prediction of shelf life ensuring the economic viability. The study will help to uncover critical factors of fermentation kinetics of *gundruk* that have remained unexplored such as the effect of biomass concentration and optimum fermentation time to give maximum lactic acid production in the final product.

## 2 MATERIALS AND METHODS

### 2.1 Fermentation experiment

Fresh *Brassica juncea* leaves were brought from the market and preliminary treatments were done viz. cleaning, washing, and removal of roots. They were then wilted in the sun for one day, crushed, and soaked in lukewarm water for 15 min. About 400 g each of crushed leaves were put into 16 jars each of 500‐ml capacity and pressed with the sterile pestle to remove excess water. The jars were then sealed hermetically and fermented at room temperature (20–25°C) for 16 days (Tamang & Tamang, 2010). Samples were taken after every 24 hr till 16th day for analyses.

### 2.2 Microbial analysis

10 g of the sample was taken and mixed with 90 ml of 0.85% w/v sterile physiological saline. After that blending was done in stomacher circulator for 5 min and the serial dilution was carried till 10^8^. Then, it was plated by pour plate method in Lactobacillus MRS Agar –M641 supplemented with 1% CaCO_3_ in triplicates. Finally, it was incubated at 30ºC in anaerobic gas pack system for 48–72 hr and the clear zone making colonies indicated the acid‐producing bacteria which were counted on the colony counter (Dewan & Tamang, 2007). Similarly, colonies of molds and yeast were examined on PDA and YM agar supplemented with 10 IU/ml benzylpenicillin and 12 µg/ml streptomycin sulfate, respectively, and incubating aerobically at 28ºC for 72 hr. Isolated colonies dependent on colony morphology were chosen randomly among the highest diluted plates. It was streaked again and the isolation media was subcultured on fresh agar plates. The microscopic examination was conducted, and the purity of the isolates was verified (Tamang & Tamang, 2010).

### 2.3 Microbial characterization and identification

A phase‐contrast microscope was used to check the cell morphology of total bacterial isolates and their motility. LAB isolates were Gram‐stained and tested for catalase production by placing a drop of 10% hydrogen peroxide solution on isolates and were preliminarily identified based on carbon dioxide production from glucose, ammonia production from arginine, growth at different temperatures (15°C, 37°C, 45°C), the ability to grow in different concentrations of sodium chloride (1%, 3%, 5%, 7%, 10%), and pH (3.9, 9.6) in MRS broth (M369, HiMedia, India) as per Tamang (2019).

### 2.4 Analytical methods

The pH was determined directly using a digital pH meter and titratable acidity was expressed as a percentage of lactic acid of the sample (AOAC, 1990). The percentage of lactic acid was converted to a gram per liter (Khadka et.al, 2010).

### 2.5 Growth and fermentation kinetics modeling

The experimental biomass was compared with the 2 different most popular models (Gompertz, and Logistic), and lactic acid production with 3 widely acceptable modeling equations ( Leudeking and Piret, Monteagudo et al., and Balannec et al.), respectively. Models in the present study were selected after observing the growth kinetics of LAB and lactic acid formation kinetics in *gundruk*. Gompertz model was selected for further study because its assumptions were similar to the results obtained and the logistic model was selected because it might explain the inhibition of the LAB population. Similarly, models that take into account parameters viz. growth‐dependent lactic acid formation and product inhibition were selected in the study. The applied microbial growth kinetics model is given in Table 1, and the product formation kinetics model is shown in Table 2.

**TABLE 1:** Microbial growth kinetics model

Model	Equations	Reference
Modified Gompertz	$\log\left(\frac{N}{N_{0}}\right)=A\exp\left\{‐\exp\left[\frac{\mu_{m}}{A}\left(\lambda ‐t\right)+1\right]\right\}$	Sharma and Mishra (2014)
Logistic	$X=\log_{10}\left(\frac{N}{N_{0}}\right)=\frac{A}{\left\{1+\exp\left[2+\left(\frac{4\mu_{m}}{A}\right)\left(\lambda ‐t\right)\right]\right\}}$	Guo et al. (2019)

**TABLE 2:** Product formation kinetics model

Model	Equations	Reference
Leudeking and Piret	$\frac{\mathrm{dP}}{\mathrm{dt}}=m\frac{\mathrm{dX}}{\mathrm{dt}}+nX$	Rajasekar, (2015)
Monteagudo et al.	$\frac{\mathrm{dP}}{\mathrm{dt}}=m\frac{\mathrm{dX}}{\mathrm{dt}}+nX\left(1‐\frac{P}{P_{\max}}\right)$	Monteagudo et al. (1994)
Balannec et al.	$\frac{\mathrm{dP}}{\mathrm{dt}}=m\frac{\mathrm{dX}}{\mathrm{dt}}+nX\left(1‐\frac{\left[\mathrm{HL}\right]}{{\left[\mathrm{Hl}\right]}_{\mathrm{inh}}}\right)$	Balannec et al. (2007)

### 2.6 Model parameters estimation

The nonlinear least‐squares regression was used to determine the kinetic parameters from nonlinear equations in microbial growth, that is, the lag period (λ), the maximum specific growth rate (μ_m_), and the log increase in population (A) reached in the culture. Similarly, the maximum concentration of lactic acid which inhibits the production of lactic acid (*P*_max_), growth‐associated coefficient (m), and non‐growth‐associated coefficient (*n*) were estimated from product formation models. The least‐square method of curve fitting was used to fit the developed models (Mavituna & Sinclair, 2008). The error between observed and predicted values was minimized by adjusting the number of iterations in the system.

Microsoft Excel 2013 was used to estimate and evaluate the parameters by fitting the experimental values to the proposed. The coefficient of determination (*R* ^2^), chi‐square (*χ* ^2^), root‐mean‐square error (RMSE), mean absolute percentage error (MAPE), and residual sum of squares (RSS) of each mathematical model were calculated, and a suitable model was chosen based on the goodness of fit with the highest value of *R* ^2^ and lowest value of *χ* ^2^, RMSE, MAPE, and RSS (Afolabi, Tunde‐Akintunde, & Adeyanju, 2015; Kaur, Arora, & Jain, 2017).

### 2.7 Statistical analysis for validation of models

To find the best suitable model to explain the fermentation behavior of any product with different conditions, statistical tools were used as described below.

### 2.8 Coefficient of determination (*R* ^2^)

It is the prediction of future outcomes based on other related information which ranges between 0 and 1. The closer it is to 1, the greater relationship exists between experimental and predicted values (Neter, 1990).

(1) $$R^{2}=\frac{{\left(\sum P_{\exp}\times P_{\mathrm{pre}}\right)}^{2}}{\sum P_{\exp}^{2}\times \sum P_{\mathrm{pre}}^{2}}$$

### 2.9 **Reduced chi‐square (** *χ* ^2^ **)**

It is the mean square of the deviations between experimental and predicted values for the models and used to evaluate the fitting agreement of each model (Ikonić et al., 2012).

(2) $$\chi^{2}=\frac{\sum_{i=1}^{N}{\left(P_{\exp,i}‐P_{pre,i}\right)}^{2}}{N‐n}$$

### 2.10 Root‐mean‐square error (RMSE)

It is the differences between values predicted by a model and the experimental values. RMSE is one of the commonly used error‐index statistics (Olyaie, Banejad, Chau, & Melesse, 2015) and is defined as:

(3) $$RMSE=\sqrt{\left(\frac{1}{N}\sum_{i=1}^{N}{\left(P_{\exp,i}‐P_{pre,i}\right)}^{2}\right)}$$

### 2.11 Residual sum of squares (RSS)

It describes the extent of the dependent variable's variation the model did not explain. It is a variation that remains after subtracting from the total variation (Fleming & Nellis, 2000).

(4) $$RSS=\sum_{i=1}^{N}{\left(P_{\exp,i}‐P_{pre,i}\right)}^{2}$$

## 3 RESULTS AND DISCUSSION

### 3.1 LAB growth

**FIGURE 1:**
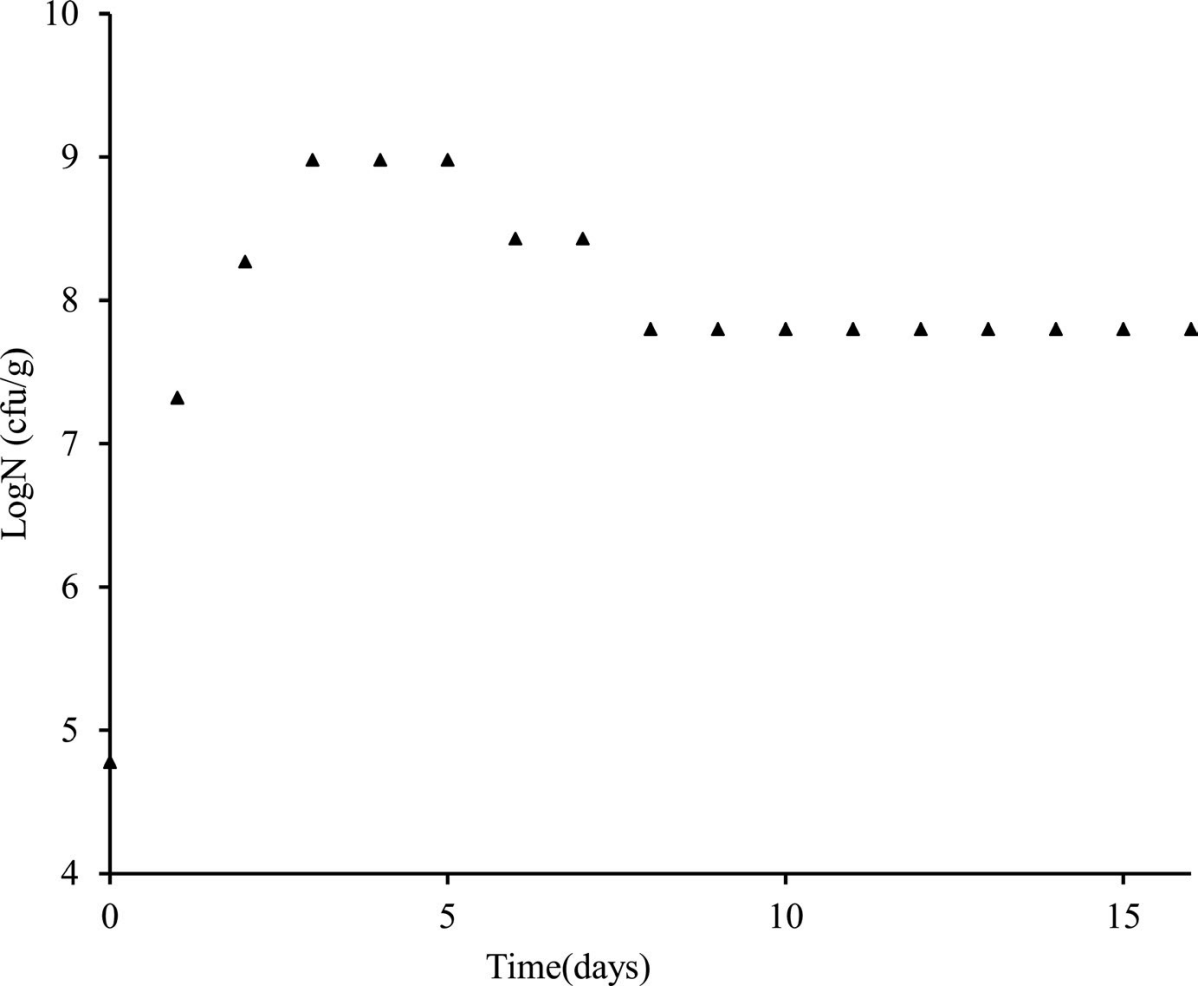
Changes in the LAB population during the natural fermentation of Gundruk

The initial population of LAB in raw *Brassica juncea* leaves was found to be 6.03 × 10^4^ cfu/g which increased significantly to the level of 9.55 × 10^8^ cfu/g till the 3rd day of fermentation. The LAB population remained constant till the 5th day and gradually decreased after 6th day to a level of 2.69 × 10^8^ cfu/g which further decreased to 6.31 × 10^7^ cfu/g and remained constant till the end of the fermentation period. Yeasts were detected only in the raw leaves and during the initial stage of fermentation. Changes in the microbial load during the fermentation of *gundruk* are shown in Figure 1.

The rapid growth of heterofermentative rods and homofermentative tetrads could be the cause of the initial exponential increase of the LAB population. Homofermentative strains of lactobacilli produce about 85% lactic acid from glucose and heterofermentative strains produce lactic acid, carbon dioxide, ethanol, and/or acetic acid in equimolar amounts (Lahtinen et al., 2011). LAB population was nearly constant during the fermentation period of 3–5 days, it might be due to the disappearance of heterofermentative lactics *(L. cellobiosus),* and vigorous growth of *L. plantarum* and other homolactics during this period. The decrease in the LAB population after 5th day might be due to the disappearance of homofermentative tetrads. Yeast and molds growth was inhibited by the end products of both homofermentative and heterofermentative LAB which interferes with the maintenance of cell membrane potential, inhibiting active transport, reducing intracellular pH, and inhibiting a variety of metabolic functions. The production of acetic acid by heterofermentive bacteria during fermentation and the controlled handling would appear to be a critical aspect in the preservation of fermented vegetables from yeasts and molds (Savard, 2002).

### 3.2 Acidity and pH

**FIGURE 2:**
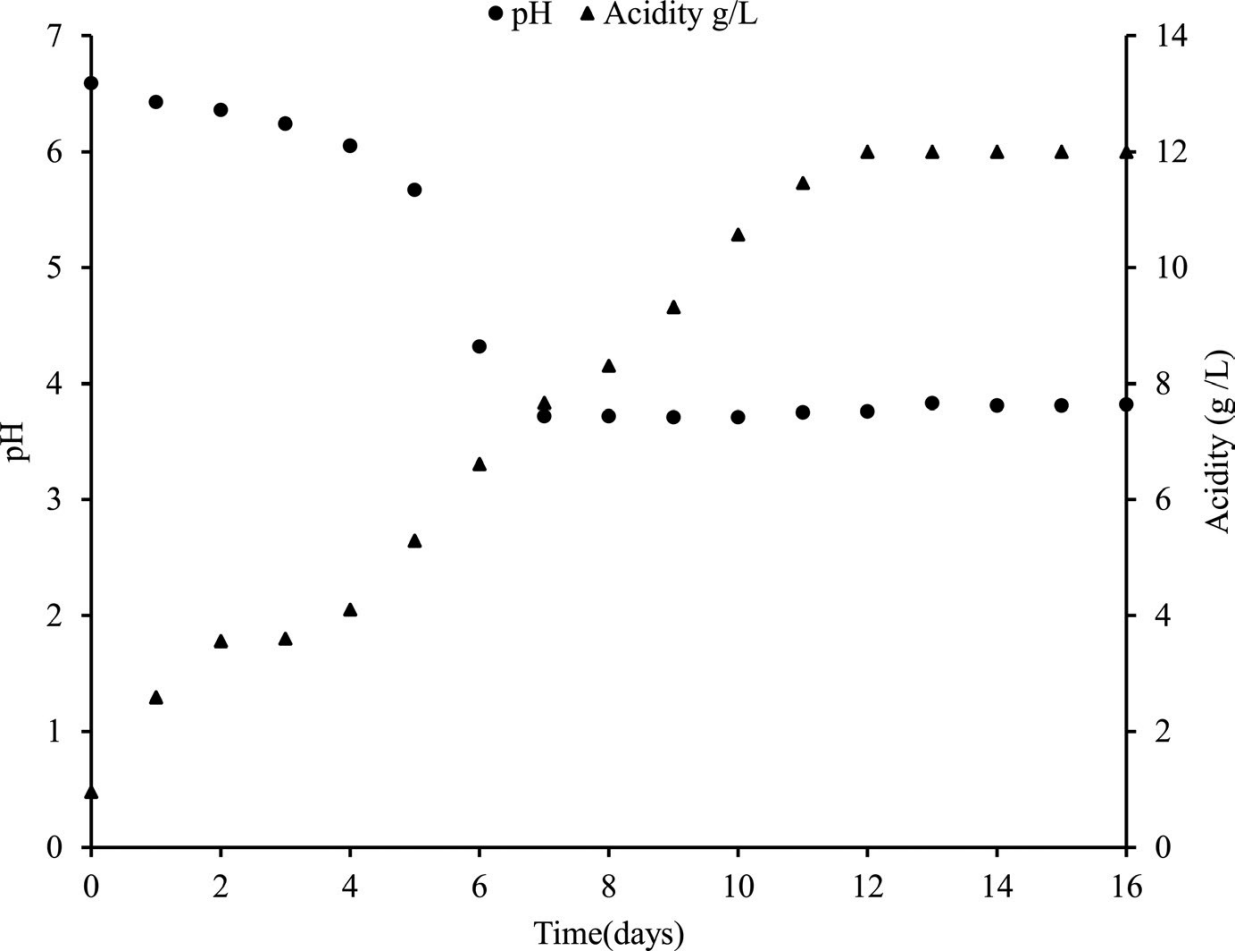
Chemical changes during natural fermentation of Gundruk

The temperature of fermenting *Brassica juncea* leaves remained around 20–22°C. The pH decreased significantly from 6.59 to 3.71, due to the formation of lactic acid by the LAB. Titratable acidity% (as lactic acid) significantly increased from an initial value of 0.095% to 1.200% at the end of the 12th day of fermentation. The chemical changes during the natural fermentation of *gundruk* are shown in Figure 2.

The lactic acid concentration increased even after the growth ceased on the 5th day of fermentation which is due to the growth of the LAB that entered the stationary phase by limitation of nutrients other than the carbon source, thereby leading to energy uncoupling of growth and lactic acid production. In the latter phase of the fermentation, increased acid production might be due to the growth of *L. plantarum* (Karki et al., 1983). Although the bacterial population is constant until the 16th day of fermentation, there is no significant increase in lactic acid concentration after the 12th day which may be due to the limitation of fermentable sugar in the substrate and also the lactic acid production resulting in end‐product inhibition. As fermentation advances with substrate utilization, the lactic acid concentration gradually increases which in turn slows down the fermentation process, including biomass, substrate utilization, and lactic acid production (Wang, Tashiro, & Sonomoto, 2015).

Shrestha, Bhattarai, & Katawal, (2012) carried out fermentation on mustard leaves at 24°C for 12 days in a glass container and found the optimum level of acidity to be 1% as lactic acid and pH of 3.9 on the 9th day. The above results showed that the acidity peaks while pH decreases the most at optimum fermentation time, which was in obedience to our findings. Dahal, Karki, Swamylingappa, Li, and Gu (2005) reported the pH value and lactic acid of *gundruk* prepared from mustard leaves to be 4.0 and 1.0%, respectively. The results are also per Tamang and Tamang (2010) who found that the pH of the fermenting substrates decreased significantly from 6.6 to 3.7 due to the growth of LAB, which converted the fermentable sugars into lactic acid.

### 3.3 Microbial growth model

Modified Gompertz and Logistic models were used to describe the growth kinetics of LAB in *gundruk* as shown in Table 1. The lag period (λ), the maximum specific growth rate (μ_m_), and the log increase in population (A) reached in the culture were estimated, and the values of these parameters and the statistical analyses are given in Table 3.

**TABLE 3:** Statistical results of growth model

Name	Parameters	*R* ^2^	*χ* ^2^	RMSE	RSS
Modified Gompertz	**µ_m_ = 0.37 hr^−1^, λ = 4.84 hr and A = 3.79**	**0.9173**	**0.3572**	**0.4095**	**1.1759**
Logistic	µ_m_ = 0.186 hr^−1^, λ = 10.30 hr and A = 3.77	0.9143	0.3874	0.4168	1.2199

From the above table, it was seen that the value of *R* ^2^ ranges between 0.9143 and 0.9173 and the lowest *χ* ^2^, RMSE, and RSS, values ranging between 0.3572 and 0.3874, 0.4095 and 0.4168, and 1.1759 and 1.2199, respectively. The value of *R* ^2^ obtained for the Logistic model is not significantly different than the Modified Gompertz model and similar values are also obtained for chi‐square, RMSE, RSS, and RDM. The Modified Gompertz model gave the best fit of the data although the results signify that both models are a good fit for experimental data.

**FIGURE 3:**
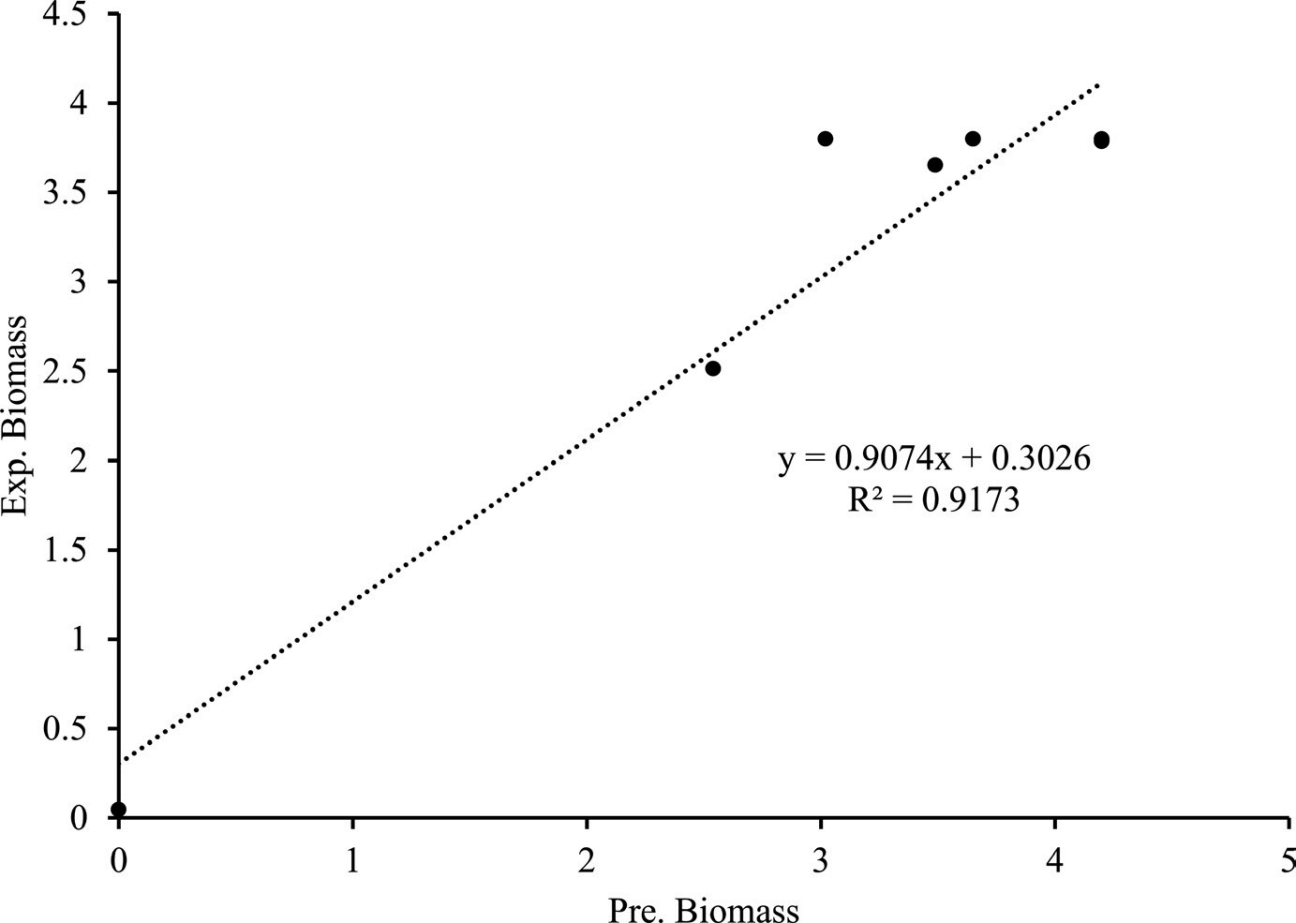
A plot of predicted and experimental biomass

Variations of experimental and predicted biomass as relative cell populations with fermentation time are given in Figure 3 that shows the biomass predicted by the Modified Gompertz model compared with the experimental data which are banded around the straight line representing data found by computation. This indicates the suitability of the mathematical model in describing the growth behavior of LAB during *gundruk* fermentation because initially *Lactobacillus cellobios* and *Pediococcus pentosaceus* grows quickly on mustard leaves (Karki et al., 1983), so that the microbial population growth followed the exponential law. Mustard leaves, on the other hand, has low sugar content but is rich in minerals and vitamins have neutral pH and thus provide a natural medium for fermentation by LAB preventing nutrient competition at the early stage (Swain, Anandharaj, Ray, & Parveen Rani, 2014). The growth kinetics of LAB during *gundruk* fermentation, as shown by the present work, agrees with the assumptions of the Gompertz equation that the rate of growth is proportional to cell mass and that the growth rate decays exponentially with time due to the inactivation of the bacteria.

Researchers have fitted the Gompertz model to *Lactobacillus plantarum* growth kinetics (Zwietering, Jongenburger, Rombouts, & van 't Riet, 1990); and, everything from plant growth, bird growth, fish growth, and growth of other animals, to tumor growth and bacterial growth (Tjørve & Tjørve, 2017). The modified Gompertz model is one of the most frequently used model for modeling growth in several bacteria and is currently one of the most common model in microbial growth (Buchanan, 1993).

### 3.4 Product formation models

The kinetic parameters were evaluated by using the equation as shown in Table 2 in which the cell concentration is expressed in terms of the relative cell population. The results of statistical analyses undertaken to estimate the goodness of the fits on these models for *gundruk* are given in Table 4.

**TABLE 4:** Statistical results of product formation model

Model	Parameters	*R^2^*	*χ* ^2^	RMSE	RSS
Luedeking‐ Piret	m = 0.1620 and *n* = 0.0099	0.9898	0.4058	0.3787	1.5775
Monteagudo *et al*.	**P_max_ = 116.544 g/L, m = 0.1104 and *n* = 0.0042**	**0.9907**	**0.3595**	**0.3601**	**1.4391**
Balannec *et al*.	(HL)_inh_ = 21.4864 g/L, m = 0.0348 and *n* = 0.0153	0.9469	2.1346	0.8622	7.4409

**FIGURE 4:**
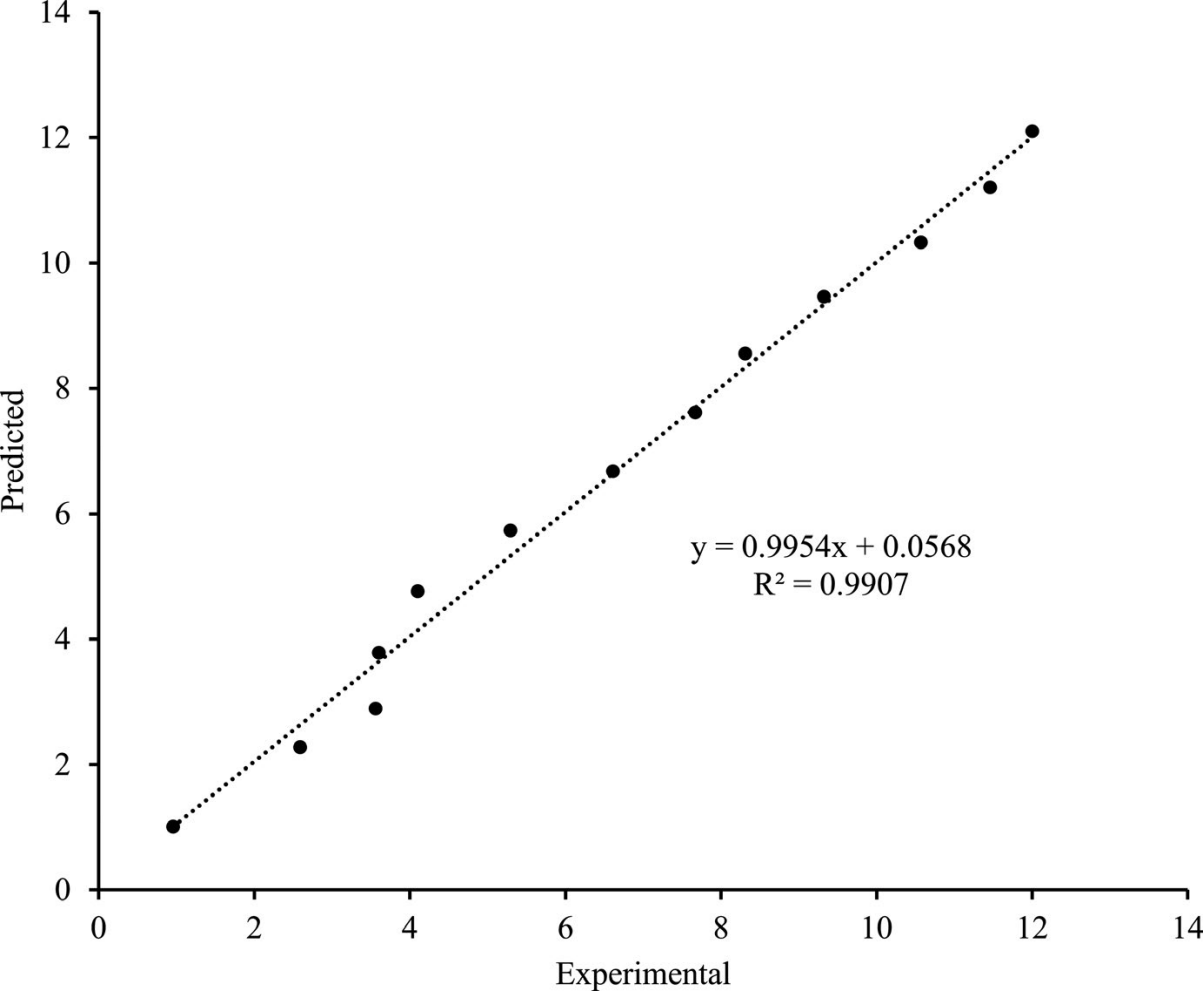
Predicted and experimental lactic acid concentration by Monteagudo et al. model

In all cases, the values of *R* ^2^ for the models are greater than the acceptable threshold of 0.80 which indicates a good fit (Guan & Yao, 2008). It was seen that the value of the coefficient of determination ranges between 0.9907 and 0.9469 and the *χ* ^2^, RMSE, and RSS values ranging between 2.1346 and 0.3595, 0.8622 and 0.2667, and 7.4409 and 0.7123, respectively. Based on results, Monteagudo et al. model fulfill all the criteria for the goodness of fit describing the lactic acid production behavior during *gundruk* fermentation. The predicted and experimental lactic acid concentration by Monteagudo et al. model is provided in Figure 4.

The kinetic parameters viz. growth‐associated coefficient "m" of 0.1104 and non‐growth‐associated coefficient "n" of 0.0042 were obtained by fitting the experimental data in Monteagudo et al. model using the nonlinear least‐squares reveals that the kinetics of lactic acid production in *gundruk* fermentation is a mixed type (Mavituna & Sinclair, 2008), that is, it follows both growth‐associated product formation kinetics and non‐growth‐associated production formation kinetics. In other words, lactic acid was produced during the growth and stationary phases of the microorganisms. Since the value of m is dominating over n, the lactic acid production kinetics is more growth associated. According to this model, the lactic acid‐producing capability of the bacteria was completely inhibited at a lactic acid concentration of 116.544 g/L (*P*_max_), which is per the result obtained by Giraud, Lelong, and Raimbault (1991).

### 3.5 Comparison of parameter values

The obtained values were compared with the results obtained from a similar and different substrate as shown in Table 5.

**TABLE 5:** Parameters for lactic acid fermentation on similar or different substrates

Parameter	LAB	LP	LH	LD	LB	LP
	(BJL)	(VJ)	(WP + YE)	(BM)	(Gowe)	
References	Current Work	Sharma and Mishra (2014)	Bouguettoucha, Balannec, Nacef, and Amrane (2011)	Monteagudo et al. (1994)	Munanga et al. (2016)	
μ_max (hr_^‐1^_)_	**0.37 **	0.53	0.63	0.831	0.22	0.28
λ (hr)	**4.84 **	2.72	_	_	1.6	1.2
A	**3.79**	3.07	_	_	_	_
m	**0.1104**	0.75	2.68	0.235	4.4	1.17
*n*	**0.0042**	0.022	0.422	0.087	2.8	5.6
P_max (g_^l‐1^_)_	**116.5 **	_	_	57	_	_

The maximum specific growth rate (μ_m_) in the present investigation was lower than previously published values for other lactic acid bacteria except the specific growth rate obtained by Munanga, Loiseau, Grabulos, and Mestres (2016). Similarly, the maximum concentration of lactic acid which inhibits the production of lactic acid (*P*_max_) was significantly higher than the value obtained by Monteagudo et al. (1994). Likewise, the lag period (λ) was significantly more than previously published values which might be due to the initially very low LAB population on the mustard leaf. The log increase in population (A) is almost following the value obtained by Sharma and Mishra (2014), since the substrate used is similar. Contrastingly, the values of growth‐associated coefficient "m" and non‐growth‐associated coefficient "n" were significantly lower in this study than previously published values. The non‐growth‐associated and growth‐associated mechanism is reported to vary with the substrate, product, temperature, and pH at optimal conditions (Roy, 1987).

## 4 CONCLUSION

*Brassica juncea* leaves were used to prepare *gundruk* by natural fermentation under an anaerobic environment at room temperature for 16 days. The exponential increase in the LAB population was significant until 3rd day detecting at the level of almost 9.55 × 10^8^ cfu/g. The LAB population after 3rd day gradually decreased and remained constant after 8th day at a level of 6.31 × 10^7^ cfu/g until the end of the fermentation period. The pH of the fermenting substrates decreased significantly from 6.59 to 3.71, while the titratable acidity increased by about 12.58 times, from an initial value of 0.095% to 1.2% at the end of the 12th day of fermentation. Modified Gompertz model gave a better fit for cell growth and multiplication while the Monteagudo et al. model suited well for lactic acid production. It was noticed that lactic acid production by LAB was mixed type and the growth‐associated coefficient "m" has been dominating over the non‐growth‐associated coefficient "n." The model could adequately describe the biochemical changes during LAB growth in the vegetable media and may be useful for controlling the growth and lactic acid production kinetics in *gundruk* fermentation.

## CONFLICTS OF INTEREST

The authors declare that they do not have any conflict of interest.

## ETHICAL STATEMENT

Ethical Review: This study does not involve any human or animal testing.
